# Effect of spring nitrogen fertilization on bearing and branching behaviors of young apple trees

**DOI:** 10.1371/journal.pone.0285194

**Published:** 2023-05-04

**Authors:** Martin Mészáros, Hana Hnátková, Patrik Čonka, Tomáš Lošák, Jan Náměstek

**Affiliations:** 1 Department of Technology, Research and Breeding Institute of Pomology Holovousy Ltd., Hořice, Czech Republic; 2 Mendel University in Brno, Brno, Czech Republic; Bahauddin Zakariya University, PAKISTAN

## Abstract

The total aboveground biomass production, nutritional status, bearing and branching behaviors of the central leader and one year old shoots of young apple trees have been analyzed. The shoots were further characterized according to the length, shoot demography, and the production of terminal and lateral flowers. All the characteristics are described in connection with nitrogen supply and cultivar. Nitrogen represents one of the major macronutrients involved in the growth and development of the fruit trees. The understanding of the effect of nitrogen supply for flower bud formation can be further improved by detailed analyses of tree architecture. While the biomass production was cultivar specific, the trees within particular cultivar were characterized by almost similar growth with respect to the nitrogen supply. Cultivar ´Rubinola´ exhibited similar branching pattern, but higher vigor than ´Topaz´. As a result of higher apical dominance, ´Rubinola´ produced higher proportion of long shoots, but a lower quality of short shoots than ´Topaz´. Consequently, cultivar ´Rubinola´ produced only few terminal flowers on short shoots and lateral flowers dominantly in the distal zone, while ´Topaz´ was characterized by intensive terminal flowering, but the lateral flowers were more abundant in the median zone. Even a lower dose of spring nitrogen improved the flower bud formation on both terminal and lateral positions extending the flowering zone on one-year-old shoots. This further changed the branching and bearing behavior of the apple trees, which particularly allows to optimize their fertilization management. However, this effect appears to be further regulated by mechanism connected with apical dominance.

## Introduction

Apples represent an important horticultural crop grown throughout the world because of its high nutritional and economic value [1]. Their growth and productivity characteristics are partly genetically determined [2, 3]. This is particularly connected with their branching patterns and fruiting habits [4] that further determine their agricultural value [5]. The bearing and branching behavior can be expressed via different bud composition along the shoots resulting from differentiation and organogenetic activity of the terminal and axillary meristems [6]. Consequently, the different rate of shoot growth leads to a production of various shoot types [7]. The flowers in apple trees are located in terminal position of shoot axes forming so called mixed buds [8]. These buds can be located both at terminal and axillary positions along the parent shoot forming spurs with leaf rosette and flower cluster. The relationship between the bud position and fate along the annual shoots determines the branching and flowering patterns [2, 9] that are consequently important for the fruit tree orchard management [10, 11].

Optimal tree nutrition is one of the main prerequisites for high yields of good quality fruits and desired vegetative tree growth [12]. Nitrogen (N) is often regarded as the most important mineral nutrient improving production of many agricultural crops worldwide. It is contained in enzymes, vitamins, in the chlorophyll molecule, involved in nucleic and amino acid synthesis, and protein production. Consequently, N is important for cell division and growth of young tissues, e.g., buds, flowers, leaves, and shoots [13]. On the other hand, excessive nitrogen inputs in agriculture represents one of the major environmental concerns. The main environmental problems associated with nitrogen fertilizers are ammonia (NH_3_) emissions and leaching of nitrate (NO_3_^-^) compounds to water. Emissions of NH_3_ are largely responsible for the acidification and eutrophication of nitrogen-limited ecosystems [14]. To minimize the highly negative effects of NH_3_ emissions or NO_3_^-^ leaching into the environment, it is thus also important to calculate an appropriate nitrogen dose of fertilizers. Two major sources of N contribute to vegetative tree growth and reproduction: (i) root N uptake and (ii) internal N cycling [15–17]. Approximately 50% of the nitrogen used for new growth of fruit trees in spring was recognized to be remobilized from the N reserves [17, 18]. For vegetative growth, the N reserves can provide the majority of N required for spur growth and up to half of N required for shoot growth [19]. The following growth is further maintained by the rapid root uptake rising the importance for new N supply [20]. High N supply tends to favor vegetative over reproductive growth. This is usually associated with different factors affecting the flower bud initiation and differentiation. Conversely, a low N supply can be associated with poor flower differentiation [21, 22]. Many studies reports the influence of nitrogen on total vegetative growth, yield response, and fruit quality of different perennial crops [15, 23, 24]. However, the knowledge about the N effect on architectural development of fruit trees underlying the link between flower bud formation and the nitrogen source and supply remain scarce [25, 26]. Our detailed analysis of shoots morphology sheds more light on the link between N supply and bearing and branching behavior of the apple trees. Moreover, this study allow us to consider the morphological variability linked with the N source reduction.

## Material and methods

### Plant material and orchard management

The trial was conducted on one-year-old apple trees planted in spring 2016 at spacing of 3 × 2 m at Research and Breeding Institute of Pomology Holovousy Ltd. (East Bohemia—Czech Republic). The trees of three apple cultivars ´Rubinola´, ´Topaz´, and ´Golden Delicious´ grafted on MM.106 were used. The trees were pruned back to 25 cm before bud break in 2016 and left to grow with a single central leader without any further pruning to ensure natural branching. The orchard was drip irrigated in volumes meeting the 80% and 90% of the estimated crop evapotranspiration in the years 2016 and 2017, respectively. The interrows were managed as periodically tilled weed fallow and the tree rows were maintained as 1 m wide herbicide strips. The plant protection was maintained according to the principles of integrated fruit production. The protection of ´Golden Delicious´ was not fully successful in 2017 and the cultivar was moderately infested by *Venturia inaequalis*. However, cultivars ´Rubinola´ and ´Topaz´ known for their scab resistance [27] were not affected.

### Soil and environment conditions

The orchard was situated in conditions of silty loam brown soils in temperate sub-continental climate. The soil pH (pH/CaCl_2_) was 6.7 and its nutrient content was composed of 111 mg P/kg, 297 mg K/kg, 174 mg Mg/kg, and 2844 mg Ca/kg when extracted by Mehlich III [28] and analyzed by inductively coupled plasma mass spectroscopy (ICP-MS, Agilent 7900, Agilent Technologies Inc., USA). The N content in the unfertilized soils ranged usually between 5–10 mg NO_3_ + NH_4_/kg when extracted into demineralized water for Nmin and by Kjehldal method [29] for organic N and further analyzed by the continuous flow analyzer (SAN++, Skalar). Soil samples were dried at room temperature, crushed and sieved through a 0.2 mm mesh for chemical analysis.

The annual cumulative rainfall was 666 mm in 2016 and 726 mm in 2017. The history of the air temperature, rainfall, and actual evapotranspiration is presented in S1 and S2 Figs.

### Fertilization treatments

All trees were fertilized in 2016 with calcium nitrate providing 15 g N per tree to maintain the initial tree growth and moderate reserves of nitrogen for the next vegetation period. In 2017, the trees of each cultivar were gathered in three plots and fertilized with different nitrogen (N) doses: (i) untreated control, (ii) treatment with 20 g N/tree/year, and (iii) treatment with 30 g N/tree/year. The nitrogen was applied within the first 8 weeks after the petal fall periodically two times per week. As a nitrogen source, ammonium nitrate through fertigation was applied. For preventing phosphorus and potassium deficiency, 15 g of each P_2_O_5_ and K_2_O per tree were applied annually.

### Estimation of the biomass of shoots and stem

The biomass of the trees was calculated as the sum of all annual shoots volume. The particular shoot volume was estimated based on volume calculated as truncated cone:

$$V=\frac{\pi h}{3}({r_{1}}^{2}+r_{1}r_{2}+{r_{2}}^{2})$$

Where V is the shoot volume (mm^3^), h is the shoot length (mm), r_1_ is the shoot base radius, and r_2_ is the shoot top radius.

The central leader and the sylleptic shoots developed in 2016 were re-measured for the biomass estimation per tree in 2017 and added to the sum of the annual shoots biomass produced in 2017.

### Assessment of the trees nutritional status

The nutritional status of the trees was assessed through the mineral analysis of leaf dry matter and included contents of nitrogen (N), phosphorus (P), potassium (K), magnesium (Mg), calcium (Ca), boron (B), zinc (Zn), manganese (Mn), and iron (Fe). The leaf samples were collected from the middle portion of the annual shoots at the end of July 2017 at the stage of a fruit diameter of up to 70% of the final size (BBCH 77, according to Meier et al. [30]). The samples were washed in demineralized water and after evaporation of the droplets of water oven dried at 50°C to constant weight. The leaf samples were than grinded by Grindomix GM 200 to a powder. The total N content in leaves was analyzed by the continuous flow analyzer (SAN++, Skalar) after previous sulphuric acid digestion (Kjeldahl digestion method [29]). The sample (0.3 g) was mixed with 2.5 ml of mineralization mixture (96% Sulphur acid, salicylic acid, and selenium) and left for 2 hours stand over. The digestion was done at 100°C for 2 hours and after addition of hydrogen peroxide for next 2 hours at 330°C [31, 32]. The content of other mineral nutrients was determined using inductively coupled plasma mass spectroscopy (ICP-MS, Agilent 7900, Agilent Technologies Inc., USA, [33]) after previous nitric acid digestion (microwave-digestion system, Discover SPD, CEM). The sample (0.25 g) was mixed with 6 ml of 65% nitric acid and digested at 200°C for 4 min. The method was specified by CEM corporation and verified by Central Institute for Supervising and Testing in Agriculture, Czech Republic under number JPP 40033.1 [32].

### Classification and morphological description of shoots

Central leader was analyzed at the metamer scale for each tree in the two consecutive years 2016 and 2017. The branching pattern was described as succession of the axillary shoots along the central leader from the base to the top. The observed axillary shoots were classified according to their length into three types coded as 1, 2, or 3, where 1) represents short shoots up to 5 cm long, 2) medium shoots 5.1–29.9 cm long, and 3) long shoots above 30 cm long. For further information about shoot demography and variability in particular cultivar and treatment see S1 Table. The flowering intensity on these shoots was described distinctly as the terminal flowering of axillary shoots and lateral flowering of axillary shoots that occurred in spring 2018. The terminal flowering of axillary shoots was coded as 0 or 1 for no terminal flowers and terminal flower cluster present, respectively. The lateral flowering abundance of axillary shoots was coded according to the position of the flowers along axillary shoots as 0, 1, 2, or 3, where 0) represents no lateral flowers, 1) median zone with flower clusters, 2) distal zone with flower clusters, and 3) median and distal zone with flower clusters as two distinct zones or one continuous zone. For further visualization, please, see the S3–S5 Figs.

### Statistical comparison of the data depending on treatment, cultivar, and shoot type

Each cultivar included 13 trees per treatment split in five replications randomly distributed within particular plot. These conditions enabled a fully factorial design. The data for estimation of the shoot volumetric biomass, mean number of nodes, length, and internode length of the central leader, as well as the nutritional status were analyzed using two-factorial ANOVA test with treatments and cultivars as factors. Aside of the significance of the particular factor, mutual interactions were also analyzed. The variables means were further compared among cultivars and treatments jointly using one factorial ANOVA test followed by Tukey’s HSD post-hoc test. For more detail results, the cultivar effect was particularly analyzed by Tukey’s HSD post-hoc test. Prior ANOVA, normality of residuals and homogeneity of variance were tested with exact Shapiro-Wilk and Cochran-Hartley-Bartlett tests, respectively. The proportion of the sylleptic and proleptic shoots produced along the central leader and the proportion of different shoot types within the total number of proleptic shoots were analyzed using Fisher’s exact test. The presence of both, the terminal flower buds and lateral flowers according to their location, was expressed as the proportion of flowering shoots from the observed shoot populations among the treatments, cultivars, and different shoot types using the Fisher’s exact test. The results of the Tukey’s HSD test and Fisher’s exact tests were considered statistically significant at *P* < 0.05 and designated by different letters. The significance of individual factors and their mutual interactions in two-factorial ANOVA was described with following symbols: Ns. = not significant, * = significant at *P* < 0.05, ** = significant at *P* < 0.01, *** = significant at *P* < 0.001. Statistical analyses of the mentioned variables were carried out using R software [34].

## Results

### Comparison of the tree central leader characteristics, shoot demography, and shoot volumetric biomass among the cultivars and treatments

The trees of ´Rubinola´ were characterized as the most vigorous among all observed cultivars. The vigor of this cultivar was expressed as 1.39 times higher mean internode length and 1.13 times higher mean number of nodes of the central leader compared with ´Topaz´ (Table 1). ´Golden Delicious´ produced central leader internode length similar to ´Rubinola´. However, the trees of each cultivar remained similar in the central leader node number when compared among the plots later used for different treatments. This observation was linked to a higher variation in range of the node number per tree within each cultivar and treatment plot represented by 16–40 nodes. The proportion of sylleptic shoots was higher in ´Golden Delicious´ (0.029–0.055), whereas it was less in ´Topaz´ (0.002–0.018). ´Rubinola´ produced no sylleptic shoots along the central leader. The proportion of nodes with proleptic lateral shoots was in mean per cultivar higher in ´Golden Delicious´ and ´Topaz´, while it was lower in ´Rubinola´. The intensity of proleptic branching of ´Topaz´ and ´Rubinola´ tended to decrease in the trees fertilized with higher N dose.

**Table 1:** The mean number of nodes, node range, length, and internode length of the central leader per tree and the proportion of nodes with sylleptic shoots grown in 2016 and proleptic shoots along the central leader in 2017 according to cultivar and treatment. Different letters (*P* < 0.05) with the values are assigned to the statistical difference among cultivars (capital letter–one per cultivar) and treatments (small letters) in particular columns.

Cultivar	Treatment	Central leader characteristics										
		No. of nodes	CV	Range of node No.	Length (mm)	CV	Intn. Length (mm)	CV	Proportion of sylleptic branching along CL	CV	Proportion of proleptic branching along CL	CV
**Rubinola**	Control	42.8 a	A	30–61	953.3 abc	A	21.8 ab	A	0.000 e	C	0.615 c	B
	20 g N/tree	50.2 a		28–60	1136.2 ab		22.3 ab		0.000 e		0.613 c	
	30 g N/tree	51.4 a		30–68	1180.0 a		22.8 a		0.000 e		0.511 d	
**Topaz**	Control	42.2 a	B	29–56	672.7 c	C	16.5 c	C	0.006 de	B	0.690 ab	A
	20 g N/tree	42.5 a		29–57	680.8 c		15.9 c		0.002 e		0.667 abc	
	30 g N/tree	42.8 a		24–64	670.8 c		15.7 c		0.018 cd		0.653 bc	
**Golden Delicious**	Control	44.1 a	AB	35–51	888.6 bc	B	20.1 b	B	0.029 bc	A	0.707 ab	A
	20 g N/tree	47.8 a		35–61	1028.5 ab		21.7 ab		0.043 ab		0.659 b	
	30 g N/tree	46.6 a		37–61	981.7 ab		20.9 ab		0.055 a		0.721 a	
**Treatment effect**		Ns.		-	Ns.		Ns.		-		*	
**Cultivar effect**		*		-	***		***		***		***	
**Treat. × Cult.**		Ns.		-	Ns.		Ns.		-		-	

ns. = not significant,

* = significance at *P* < 0.05,

*** = significance at *P* < 0.001,

CV–cultivar effect, CL–central leader

**Fig 1:**
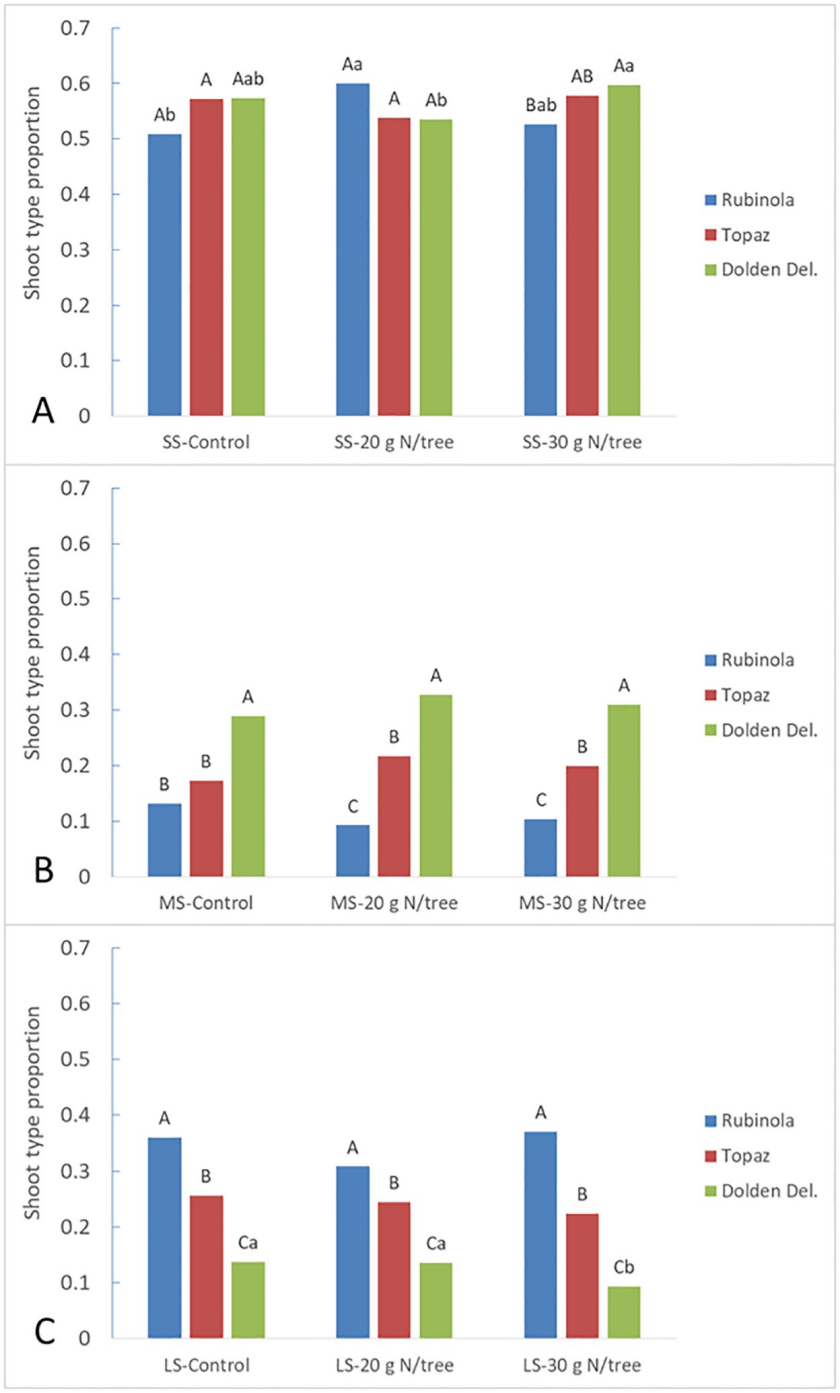
The proportion of A) SS–short shoots, B) MS–medium shoots, and C) LS–long shoots grown as proleptic shoots including terminal prolonging shoot, when compared according to cultivar and treatment in 2017. Different letters (*P* < 0.05) represents the statistical difference of the variable via the Fisher’s exact test among cultivars (capital letters) and treatments (small letters).

The short shoots represents the most extensive shoot category including 50–60% of all proleptic shoots whatever the cultivar or treatment. The cultivars differed in the proportion of the three proleptic shoot types (Fig 1), where the highest proportion of long shoots was found in ´Rubinola´ (0.35), whereas it was lower in ´Topaz´ (0.24), and the lowest in ´Golden Delicious´ (0.12). In general, the reverse was true for medium shoots. The quality of short shoots in ´Rubinola´ was lower than in other two cultivars (S1 Table). There was only little difference in shoot composition among the treatments of particular cultivars (Fig 1). ´Rubinola´ produced lower proportion of short shoots in both the trees treated as Control and with 30 g N/tree compared to those treated with 20 g N/tree. ´Golden Delicious´ produced a larger number of short shoots and less long shoots in trees treated with higher nitrogen dose.

Table 2 shows that the initial shoot biomass was the highest in ´Rubinola´ (0.075–0.099 mil. mm^3^) and the lowest in ´Topaz´ (0.054–0.057 mil. mm^3^) when produced by the trees in 2016. The trees in Control plot before the N treatment produced less biomass in ´Rubinola´ and ´Golden Delicious´ compared to that determined for both treatments with nitrogen. In 2017, the shoot biomass was again the highest in ´Rubinola´ trees and the lowest in ´Golden Delicious´. This was due to a higher annual production of shoot biomass in the former cultivar. Despite of a certain trend, an increase in biomass in these two cultivars treated with a higher N dose was not significant in comparison with the Control.

**Table 2:** The absolute volumetric biomass of shoots produced by the trees in the years 2016, 2017 and the difference between 2016 and 2017 for particular cultivars and treatments. Different letters (*P* < 0.05) with the values are assigned to the statistical difference among cultivars (capital letter) and treatments (small letter) in particular columns.

Cultivar	Treatment	Biomass 2016 (mil. mm^3^)	CV	Biomass 2017 (mil. mm^3^)	CV	Biomass 2017–2016 (mil. mm^3^)	CV
**Rubinola**	Control	0.075 b	A	0.546 ab	A	0.471 a	A
	20 g N/tree	0.098 a		0.602 ab		0.504 a	
	30 g N/tree	0.099 a		0.666 a		0.567 a	
**Topaz**	Control	0.056 d	B	0.489 abc	B	0.433 ab	B
	20 g N/tree	0.054 d		0.451 bcd		0.397 abc	
	30 g N/tree	0.057 d		0.460 abc		0.404 abc	
**Golden Delicious**	Control	0.046 e	B	0.245 d	C	0.200 d	C
	20 g N/tree	0.066 c		0.307 cd		0.241 cd	
	30 g N/tree	0.064 c		0.322 cd		0.258 bcd	
**Treatment effect**		-		Ns.		Ns.	
**Cultivar effect**		***		***		***	
**Treat. × Cult.**		-		Ns.		Ns.	

ns. = not significant,

*** = significance at *P* < 0.001.

CV–cultivar effect

### Comparison of the axillary shoots flowering intensity among cultivars, treatments, and shoot types

**Fig 2:**
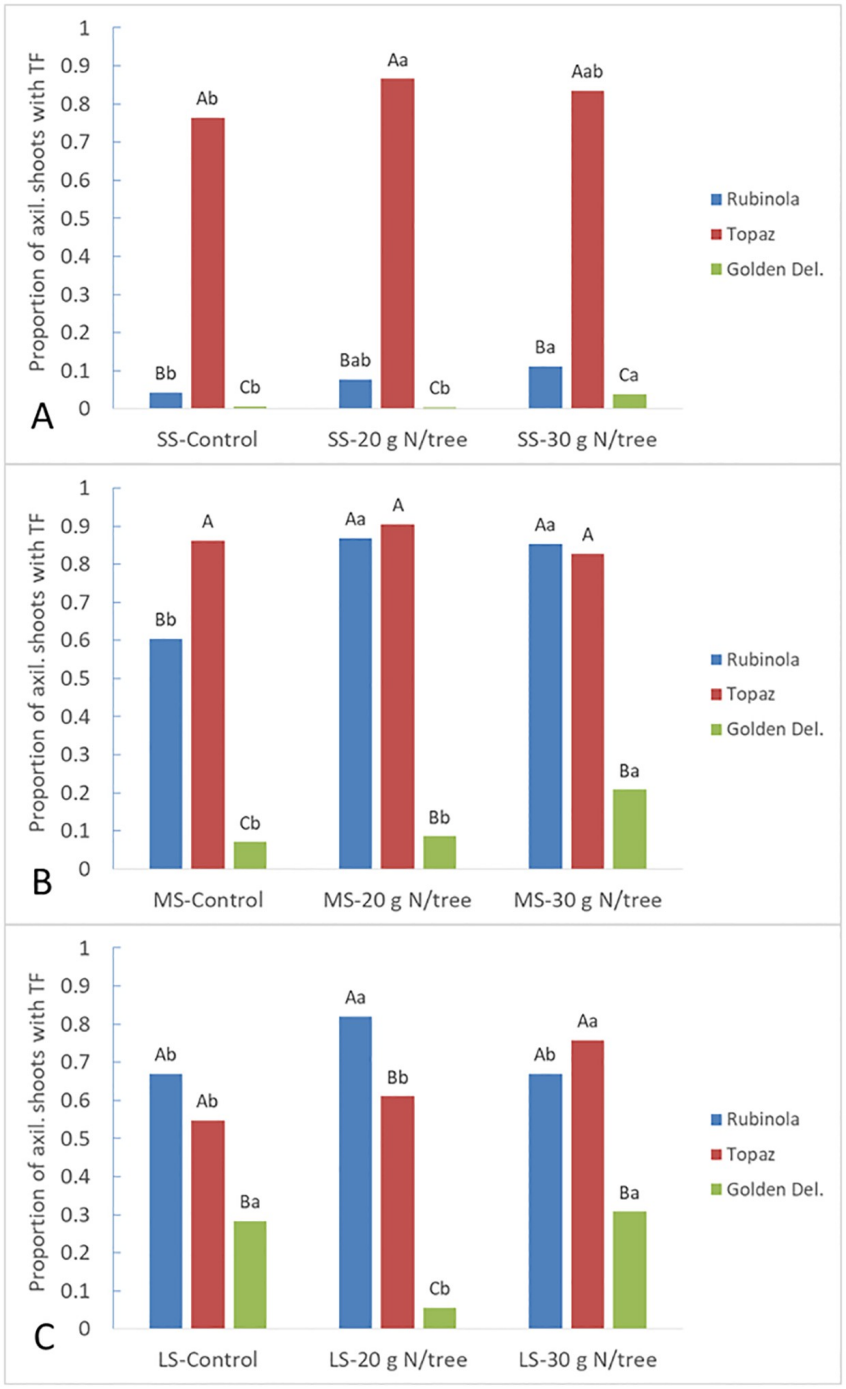
The proportion of axillary shoots with terminal flowers according to the cultivar ´Rubinola´, ´Topaz´, and ´Golden Delicious´ and treatment on A) short (SS), B) medium (MS), and C) long shoots (LS) in 2018. Different letters (*P* < 0.05) represents the statistical difference of the variable via the Fisher’s exact test among cultivars (capital letters) and treatments (small letters).

Fig 2 reveals that ´Rubinola´ was characterized in 2018 by a very low (up to 0.11) proportion of short shoots with terminal flowering and moderate to high proportion of medium shoots and long shoots with terminal flowering (0.61–0.87). ´Topaz´ produced a proportion of medium shoots and short shoots with terminal flowering (0.76–0.91) higher than that on long shoots (0.55–0.76). The overall proportion of terminal flowering in ´Golden Delicious´ shoots was low with the highest values in the long shoots of up to 0.31. ´Topaz´ had proportion of axillary shoots with terminal flowers especially on short shoots 10.6–48.1 times higher than ´Rubinola´ and ´Golden Delicious´, respectively.

The cultivars differed in the reaction on the fertilization treatments (Fig 2). Terminal flowers production on short shoots and medium shoots increased in both ´Rubinola´ plots treated with nitrogen, whereas on long shoots only when treated with 20 g N/tree. The proportion of short shoots with terminal flowers in ´Topaz´ was higher at lower N dose, whereas on long shoots it was higher at higher N dose compared to untreated Control. ´Golden Delicious´ produced higher proportion of short shoots and medium shoots with terminal flowers only when treated with a higher N dose.

**Fig 3:**
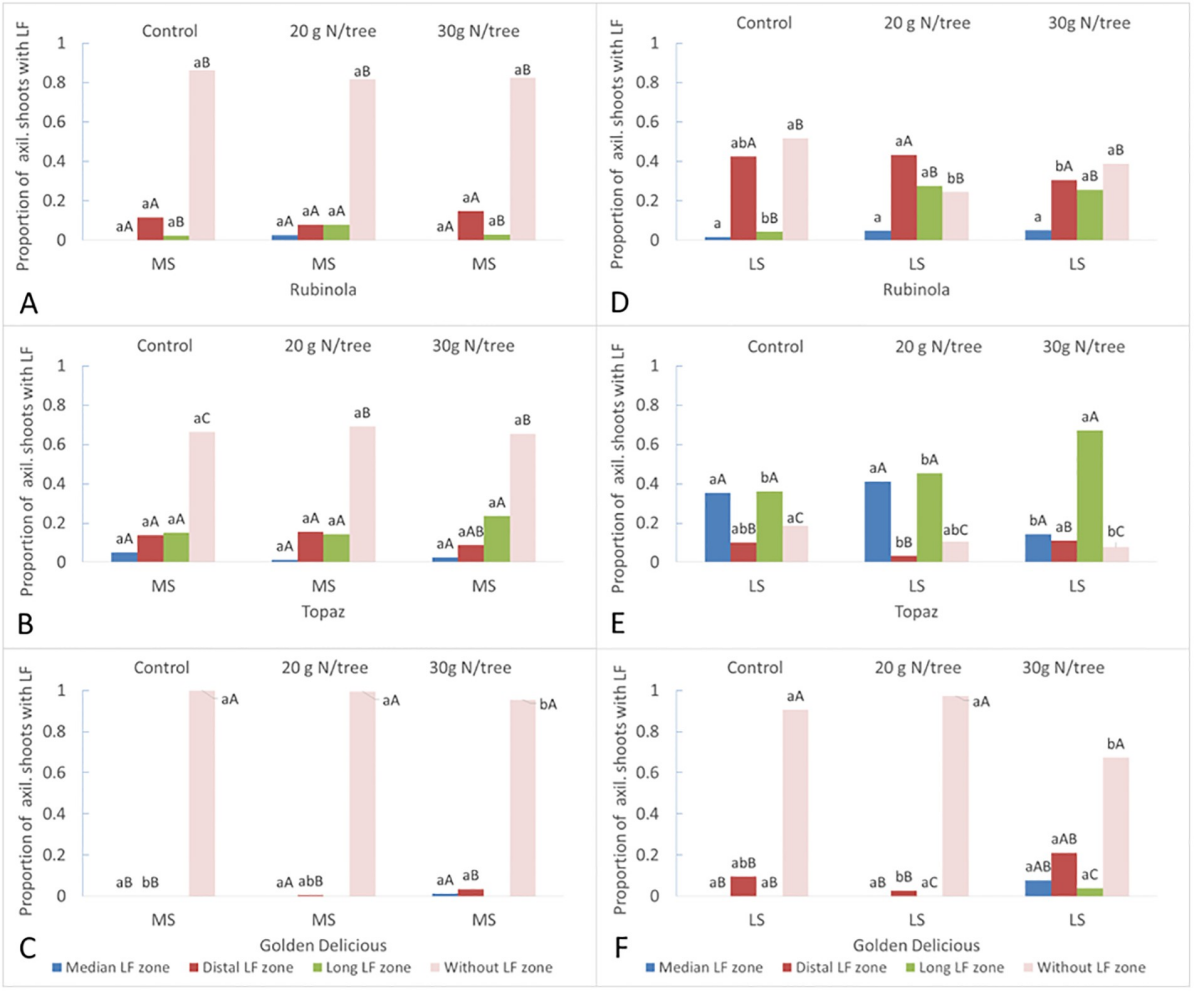
The proportion of axillary shoots with different pattern of lateral flowering according to cultivar Rubinola (A and D), Topaz (B and E), and Golden Delicious (C and F), treatment and shoot type -medium shoots (A, B, and C) and long shoots (D, E, and F) in 2018. Different letters (*P* < 0.05) represents the statistical difference of the variable via the Fisher’s exact test among cultivars (capital letters) and treatments (small letters).

None of the cultivars formed any lateral flower zone along the short shoots. The proportion of axillary shoots with lateral flower zone increased from medium shoots toward long shoots (Fig 3A–3F). The cultivars differed in the total proportion of shoots with lateral flower zone as well as in the proportion of shoots with the specific lateral flower zone position along one-year-old shoots. ´Rubinola´ exhibited a lower proportion of medium shoots (up to 0.18) and long shoots (0.48–0.76) with lateral flower zone than ´Topaz´ in which the values ranged between 0.31–0.35 and 0.82–0.92 for medium shoots and long shoots, respectively. While ´Rubinola´ produced medium and long shoots more frequently with distal lateral flower zone (up to 84% and 88%), the reverse was true in shoots of ´Topaz´, especially in long shoots containing up to 96% of lateral flowers as median or long zone jointly.

The proportion of medium shoots with lateral flower zone remained similar for each cultivar no matter of the treatments. ´Rubinola´ increased the proportion of long shoots with long lateral flower zone when treated with nitrogen. However, the cultivar tended to further decrease the proportion of long shoots with distal lateral flower zone increasing the proportion of non-productive shoots at higher N dose. The trees of ´Topaz´ produced a higher proportion of long shoots with long lateral flower zone (0.67) when treated with 30 g N/tree compared to both Control (0.36) and trees treated with the lower N dose (0.45). Similar tendency can be observed in Topaz medium shoots, which was linked with decrease in both, the median and distal zones on behalf of the long lateral flowering zone. ´Golden Delicious´ was overall low in the production of shoots with lateral flowers preferring the distal zone position, but usually did not exceeded a proportion of 0.1. The only exception was found in the trees treated with 30 g N/tree where approximately one third of shoots produced lateral flower zone.

### Comparison of the nutritional status in leaves of the apple trees according to the cultivar and treatment

The mineral content in apple leaves is presented in Table 3 for macronutrients and Table 4 for micronutrients. The highest contents of nitrogen and phosphorus, but a lower magnesium content in leaf dry matter, were found in ´Rubinola´ comparing to ´Topaz´ and ´Golden Delicious´. Cultivar ´Topaz´ had higher manganese contents than the other two cultivars and higher calcium content in leaf than ´Rubinola´. ´Golden Delicious´ was characterized by a lower potassium and boron contents in leaf dry matter in contrast to both ´Rubinola´ and ´Topaz´. The treatment effect was observed in the N, P, and Mg contents only. The N content in cultivars ´Rubinola´ and ´Topaz´ was higher in trees treated with 30 g N/tree (2.60% and 2.40%) than in untreated control (2.46% and 2.28%) and in the case of ´Topaz´ also at the lower dose treatment (2.26%). The highest N content in ´Golden Delicious´ was found after the treatment with the lower dose (2.35%). The P content in leaf dry matter always appeared to be higher in control treatments whatever the cultivar was. The Mg content was higher after the full N dose treatment in ´Rubinola´ and ´Golden Delicious´. However, this was not true for ´Topaz´, where the Mg content was the highest in control (0.190%) and the lowest in trees receiving 20 g N/tree (0.155%). Similar tendency was found with iron content in cultivar ´Topaz´. The N, Mg, and Fe content indicated interactions among cultivars and treatments.

**Table 3:** Macronutrients content in leaf dry matter of the three apple cultivars at BBCH 77 according to the fertilization treatments in 2017. Different letters (*P* < 0.05) with the values are assigned to the statistical difference among cultivars (capital letters) and treatments (small letters) in particular columns.

Cultivar	Treatment	Macronutrients in % of dry matter									
		N	CV	P	CV	K	CV	Mg	CV	Ca	CV
**Rubinola**	Control	2.46 bc	A	0.220 a	A	2.01 a	A	0.130 e	B	0.88 b	B
	20 g N/tree	2.49 ab		0.190 abc		2.16 a		0.135 de		0.89 ab	
	30 g N/tree	2.60 a		0.195 abc		1.98 a		0.150 cde		1.00 ab	
**Topaz**	Control	2.28 ef	B	0.200 ab	B	2.26 a	A	0.190 abc	A	1.14 a	A
	20 g N/tree	2.26 ef		0.160 bc		2.01 a		0.155 cde		0.95 ab	
	30 g N/tree	2.40 bcd		0.175 abc		2.00 a		0.175 bcd		1.08 ab	
**Golden Delicious**	Control	2.23 f	B	0.170 bc	B	1.88 a	B	0.190 abc	A	0.90 ab	AB
	20 g N/tree	2.35 cde		0.160 bc		1.85 a		0.215 ab		0.95 ab	
	30 g N/tree	2.29 def		0.150 c		1.75 a		0.225 a		1.03 ab	
**Cultivar effect**		***		***		*		***		*	
**Treatment effect**		**		**		Ns.		*		Ns.	
**Treat. × Cult.**		**		Ns.		Ns.		*		Ns.	

ns. = not significant,

* = significance at *P* < 0.05,

** = significance at *P* < 0.01,

*** = significance at *P* < 0.001.

CV–cultivar effect

**Table 4:** Micronutrients content in leaf dry matter of the three apple cultivars at BBCH 77 according to the fertilization treatments in 2017. Different letters (*P* < 0.05) with the values are assigned to the statistical difference among cultivars (capital letters) and treatments (small letters) in particular columns.

Cultivar	Treatment	Micronutrients in ppm of dry matter					
		B	CV	Zn	Mn	CV	Fe
**Rubinola**	Control	46.4 a	A	14.9 a	36.9 bcd	B	102.5 ab
	20 g N/tree	44.0 a		14.0 a	33.4 bcd		83.4 b
	30 g N/tree	45.6 a		20.8 a	34.4 bcd		88.5 ab
**Topaz**	Control	46.7 a	A	21.1 a	45.6 a	A	97.9 ab
	20 g N/tree	36.4 a		15.8 a	40.8 abc		119.0 a
	30 g N/tree	41.8 a		16.0 a	41.4 ab		88.8 ab
**Golden Delicious**	Control	36.5 a	B	16.1 a	31.2 d	B	103.8 ab
	20 g N/tree	37.4 a		18.0 a	32.5 cd		93.3 ab
	30 g N/tree	31.2 a		15.2 a	36.3 bcd		99.4 ab
**Cultivar effect**		*		Ns.	***		Ns.
**Treatment effect**		Ns.		Ns.	Ns.		Ns.
**Treat. × Cult.**		Ns.		Ns.	Ns.		*

ns. = not significant,

* = significance at *P* < 0.05,

*** = significance at *P* < 0.001.

CV–cultivar effect

## Discussion

### Characterization of the shoot growth of apple trees depending on N supply

Nitrogen supply significantly influences the trees growth and branching [25]. However, as the spring remobilization represents a major source of nitrogen for leaf growth of apple trees [20], the effect of the annual N supply on the shoot growth is likely proportional to the available N reserves. Therefore, it is not surprising that the trees woody biomass produced in the year 2017 was similar or with only a small advantage for vegetative growth among the treatments with increasing N supply. The low effect of spring nitrogen fertilization was seen also in some previous studies [24]. However, this was identified by the authors as a reaction on high fertility of the soils used for the trials, which is not our case. The rates of N fertilization among treatments started in 2017 and the occasional difference in the branching intensity along the central leader was rather connected with its node number and length. As the trees maintained similar proportion of extension shoots from the total number of nodes, higher number of outgrown buds resulted in an increased proportion of developed short shoots. The almost similar growth vigor of the apple trees of particular cultivars allowed comparison and interpretation of their nitrogen status with flowering pattern with respect to the treatments more precisely.

### Characterization of the mineral content of apple trees depending on N supply

The sufficient N content in mid-summer leaves indicated a continuous supply of this element throughout the vegetation period on silty loam soil in all treatments [35]. However, a higher content of nitrogen in leaves of N fertilized apple trees suggested a higher uptake and accumulation of this nutrient [23, 36]. It indicates that this nutrient status provides the trees with additional N resources [15, 35, 37] and allows their unlimited growth, organ development, as well as boosting of the autumn remobilization into storage tissues [20]. Nitrogen also affects the absorption and distribution of practically all other nutrients in the plant and is particularly important to the tree during flowering and fruit set [13]. In our trial, higher N supply led to an increase in Mg and decrease in P leaf content. These tendencies were in accordance with previous findings [36, 38]. However, aside of nitrogen, all other nutrients appeared to be in optimal or only slightly suboptimal contents range sufficiently covering the tree needs [39].

It is also worth of noting that the leaf nitrogen content in trees supplied with 20 g N/tree did not change compared to that in control trees. As the trees produced similar above-ground biomass but at a higher flower bud formation, the additional nitrogen could be consumed for the flower bud initiation and differentiation in addition to root growth [15]. This could explain, why this additional N cannot be tracked via the applied mineral analyses of leaves. However, the role of N in the initial phase of flower bud formation needs to be further examined and the mechanism of its transport into the bud determined via labelled N.

### Characterization of the branching and bearing behaviors of apple trees depending on cultivar and N supply

Contrasting to ´Golden Delicious´, cultivars ´Rubinola´ and ´Topaz´ featured similar branching pattern producing longer axis close to the terminal positions along the central leader with almost no tendency for sylleptic branching. However, higher ´Rubinola´ woody biomass amount was a consequence of more vigorous growth of the tree extension shoots keeping lower investments in quality of the short shoot development (Fig 1, S1 Table). Genetic determination of the branching pattern in apple trees was previously described by Costes and Guédon [2] with the differences among cultivars or ideotypes as presented by Lespinasse [4]. Nevertheless, the effect or interaction with the environmental conditions remains important [3]. In our study, the vegetative development was linked with different bearing behavior, or more specifically, flower bud distribution. While ´Rubinola´ trees produced only a few terminal flowers on short shoots, cultivar ´Topaz´ was characterized by intensive flowering on this shoot type. This is usually connected with earlier cessation of the shoot growth as more vigorous shoots tend to develop terminal buds less frequently [22]. Furthermore, ´Rubinola´ differentiated lateral flowers dominantly in the distal zone, whereas the most lateral flowers in ´Topaz´ were born in the median zone (Figs 2 and 3, and S5 Fig). Such behavior along trunk and extension lateral shoots indicate a cultivar specific reiteration in the predominant position for flower bud formation [8]. This likely corresponds with the cultivar preference for shoot resources distribution between the apical and lateral meristems growth [40, 41] controlled by the trees hormonal activity [22]. The flower bud formation is dependent on reaching critical number of leaf primordia within the bud and its required length is also genetically determined [42]. It suggests that the low investment of nutrients and carbohydrates into lateral bud development during the active vegetative growth of the shoot apex can result in failure of the flower bud formation on lateral spurs. On the contrary, the flower bud formation can start earlier, and therefore, on more basal node rank along the parent shoot in the cultivars more intensively supporting lateral bud development, i.e., with lower gradient of apical dominance.

If nitrogen becomes scarce, continuous N supply to fruit trees usually improves number and length of the developed long axillary shoots [25]. This fact may directly imply with the following bud development necessary for further flower bud formation. The spring fertilization applied within the first 8 weeks after flowering changed the flowering behavior in apple trees improving both the terminal and lateral flower bud formation. Generally, a higher production of flowers with spring N supply is in accordance with the previous findings [15, 22]. Apple tree spurs stop to grow early and the flower bud induction starts approximately 3–6 weeks after full bloom [21]. If no other signal interruptions occur [22, 42], the sufficient carbohydrates and nitrogen availability in this period most likely stimulates the differentiation process. In extension shoots, the flower bud induction starts later and the first sign of flower differentiation may be found 12–13 weeks after full bloom, long before the end of the shoot growth [43]. Supposing that the spring N supply supports the flower bud formation of apple trees, from the morphological point of view it appears to improve the development of lateral axis forming flowers in more extensive locations along the parent shoots. This is likely achieved by extension of the period with optimal nutrient supply continuously supporting the flower initiation of newly developing buds along the parent shoot. However, this process is regulated by the intensity of the apical dominance of the cultivar promoting the apical versus lateral axis development via enhanced vigor of the extension shoots [44]. In this case, a higher N supply may limit the terminal and lateral flower bud formation through increased competition by vegetative growth, as suggested by Hanke et al., [42].

## Conclusions

This study describes in more detail the effect of spring nitrogen fertilization on apple tree shoot architecture with special focus on their bearing and branching behavior. Spring nitrogen supply improves the flower bud formation on terminal as well as lateral positions via extending the flowering zone on one-year-old shoots and changing the branching and bearing behavior of the apple trees. This process is subjected to the hormonal and sink strength regulation involved in the cultivar specific apical dominance. The intensity of the apical dominance further regulates the competition between the terminal and lateral meristems consequently changing their rate of vegetative development and ability for flower bud formation. The mechanism of the nitrogen metabolism in the flower bud initiation needs to be further examined. Similar answer in bearing and branching behaviors of young apple trees on optimal as well as suboptimal conditions of nitrogen impute allows to certain reduction of the nitrogen fertilizers use in orchards.

## Supporting information

S1 Fig The daily mean air temperature at a height of 2 m and rainfall in the years 2016–2017. (TIF)

S2 Fig The daily mean potential evapotranspiration in the years 2016–2017. (TIF)

S3 Fig The flower distribution on shoots along the central leader of two years old trees of cultivars A) ´Rubinola´, B) ´Topaz´, and C) ´Golden Delicious´. (TIFF)

S4 Fig The principal zonation of the flower buds according to their terminal or lateral position along the shoots. (TIF)

S5 Fig The lateral flowers abundance according to the position of the flowers along long axillary shoots in cultivar ´Topaz´. The images show A) no lateral flowers, B) median zone with flower clusters, C) distal zone with flower clusters, and D) median and distal zone with flower clusters as two distinct zones or one continuous zone.
(TIFF)

S1 Table The mean, minimum and maximum shoot number per class (short, medium, and long shoots) along the central leader and their mean, minimum and maximum shoot length according to the cultivar, treatment and the time of shoot development (2016 –sylleptic shoots, 2017 –proleptic shoots). (DOCX)

## Abbreviations

CL: central leader
GD: ´Golden Delicious´
LS: long shoot above 30 cm
MS: medium shoots from 5.1–29.9 cm
N: nitrogen
R: ´Rubinola´
SS: short shoot up to 5 cm
T: ´Topaz´
