# Supplementary figures and images for: Effect of spring nitrogen fertilization on bearing and branching behaviors of young apple trees
Source: PLoS One. 2023 May 4;18(5):e0285194. doi: 10.1371/journal.pone.0285194 (PMC10159155; doi:10.1371/journal.pone.0285194)

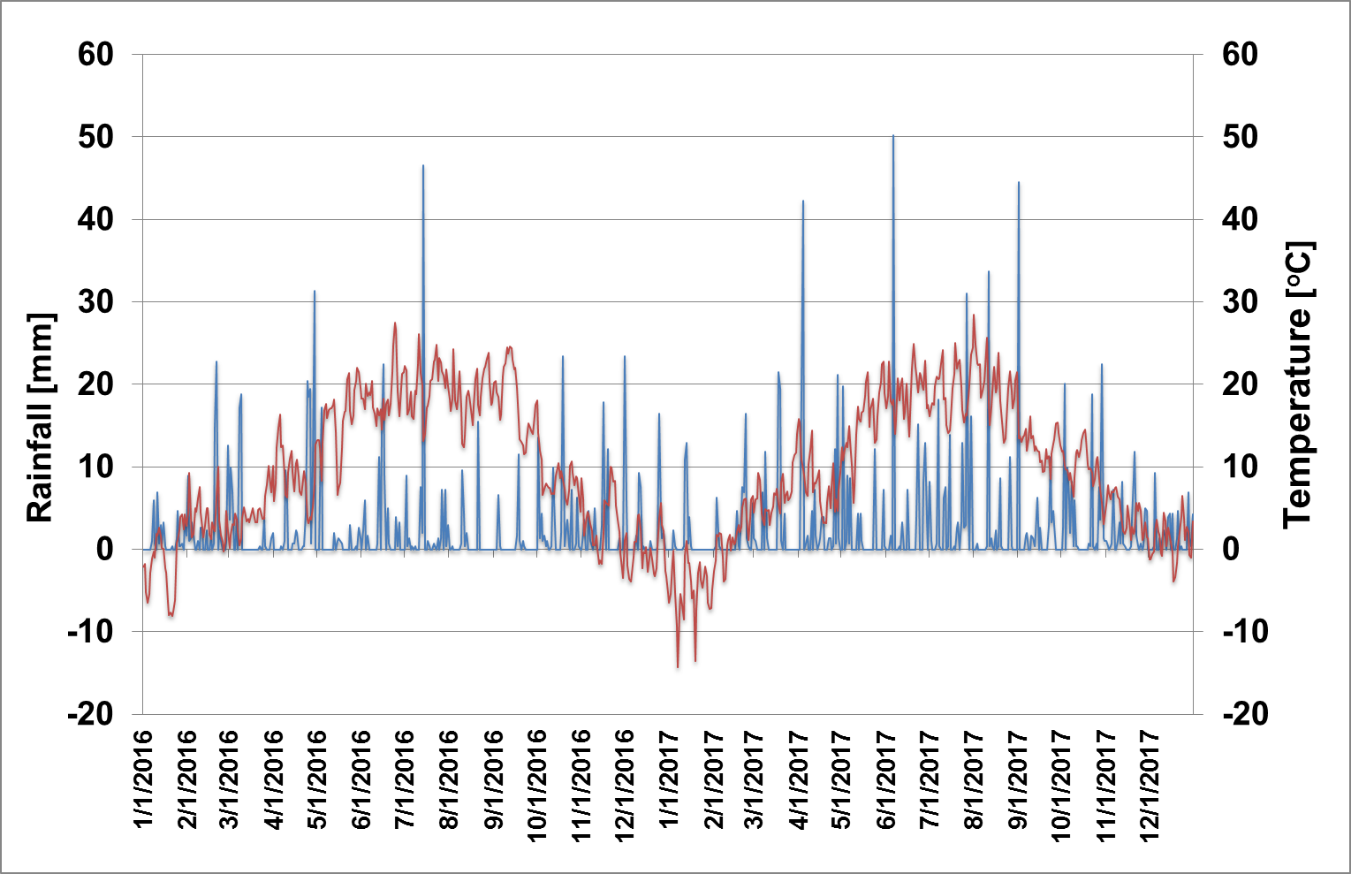

Supplement: S1 Fig — (TIF) [file pone.0285194.s001.tif]

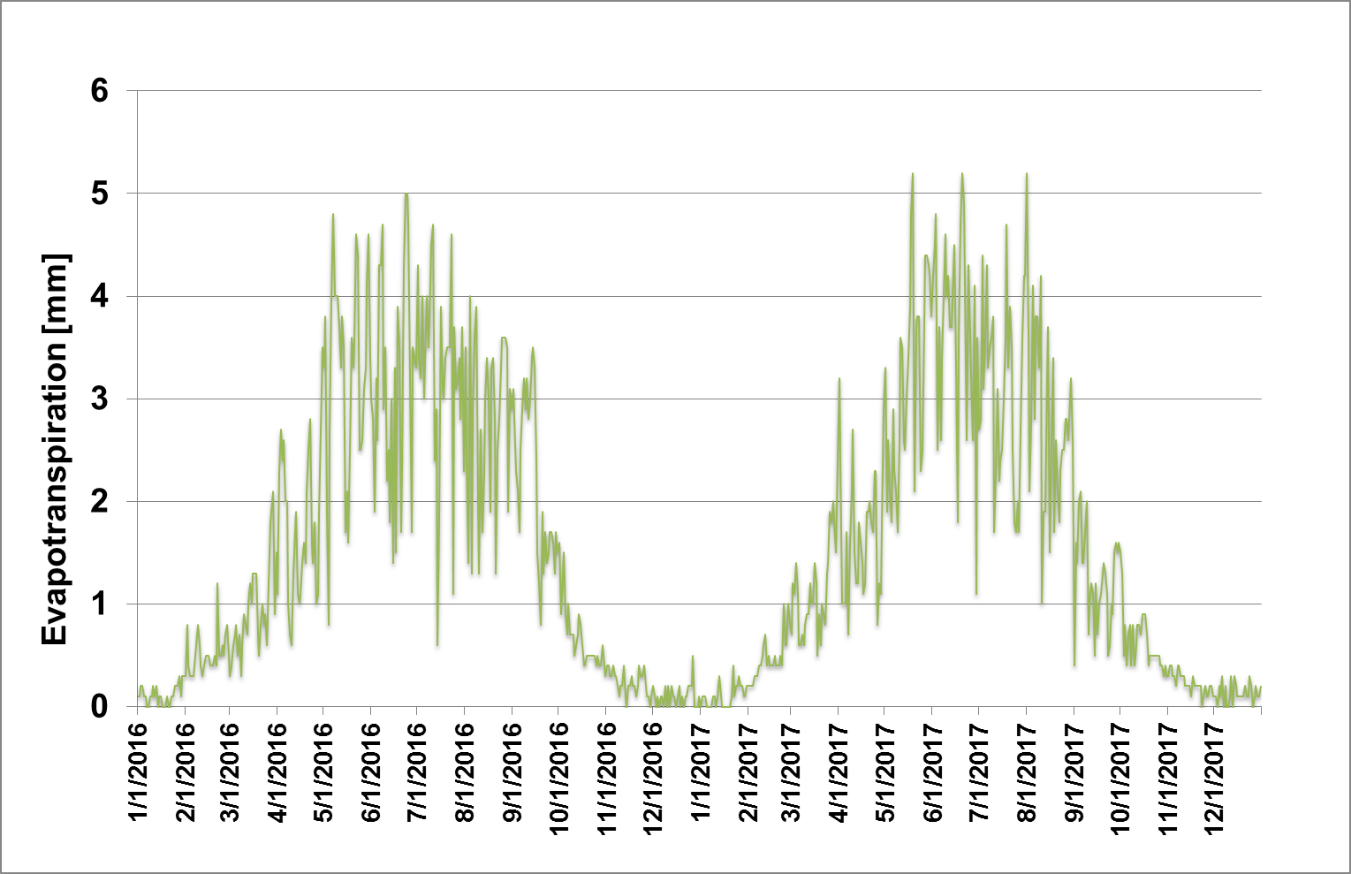

Supplement: S2 Fig — (TIF) [file pone.0285194.s002.tif]

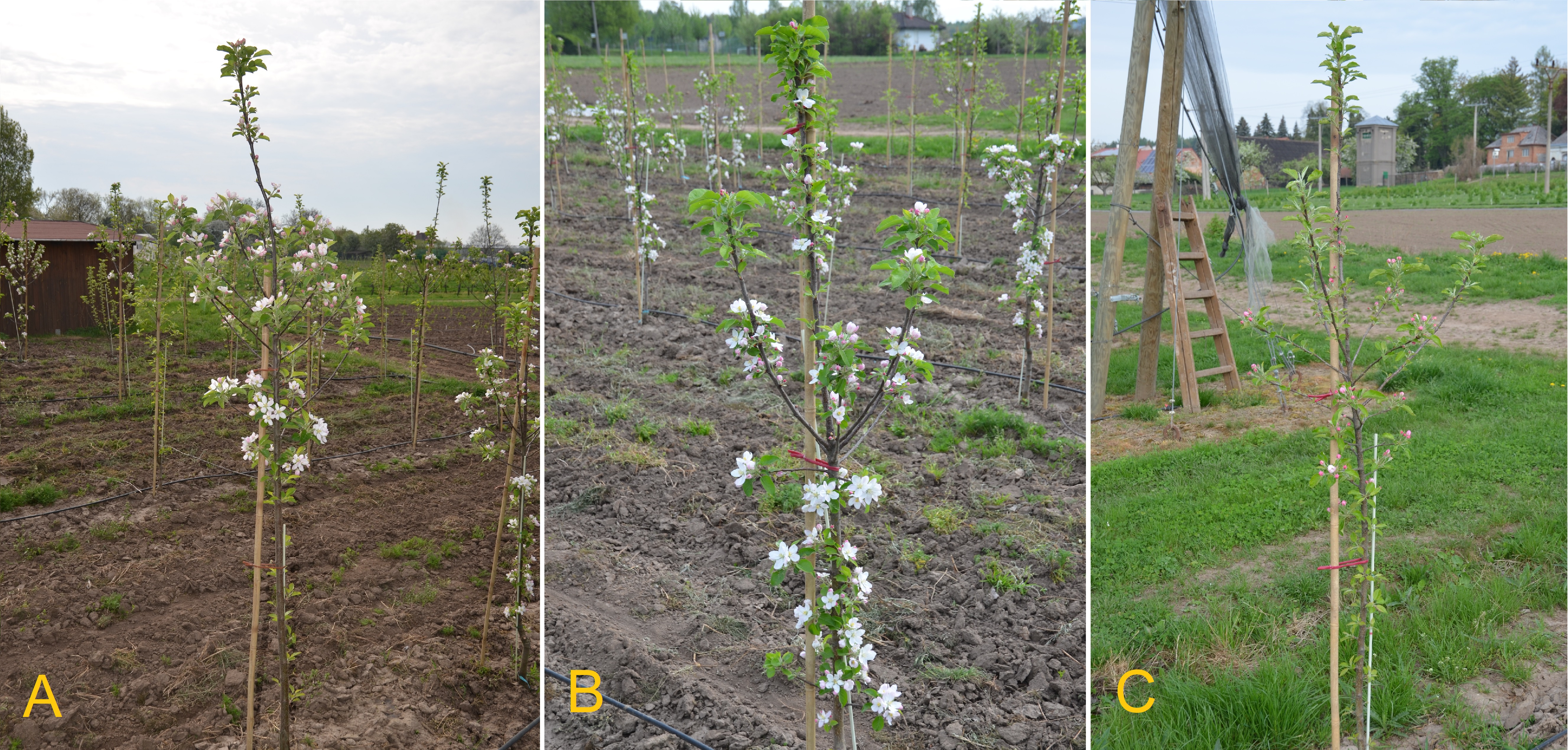

Supplement: S3 Fig — (TIFF) [file pone.0285194.s003.tiff]

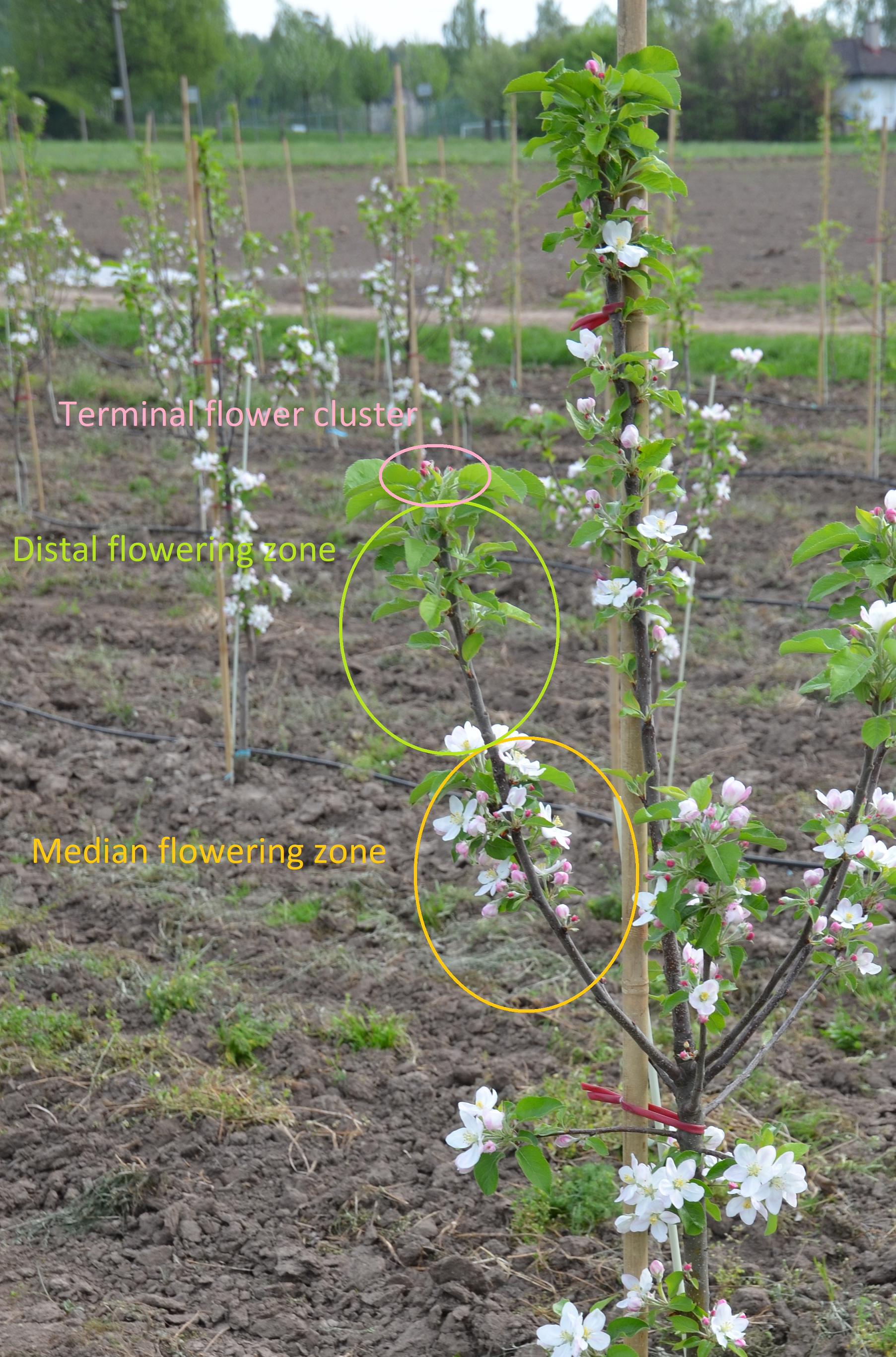

Supplement: S4 Fig — (TIF) [file pone.0285194.s004.tif]

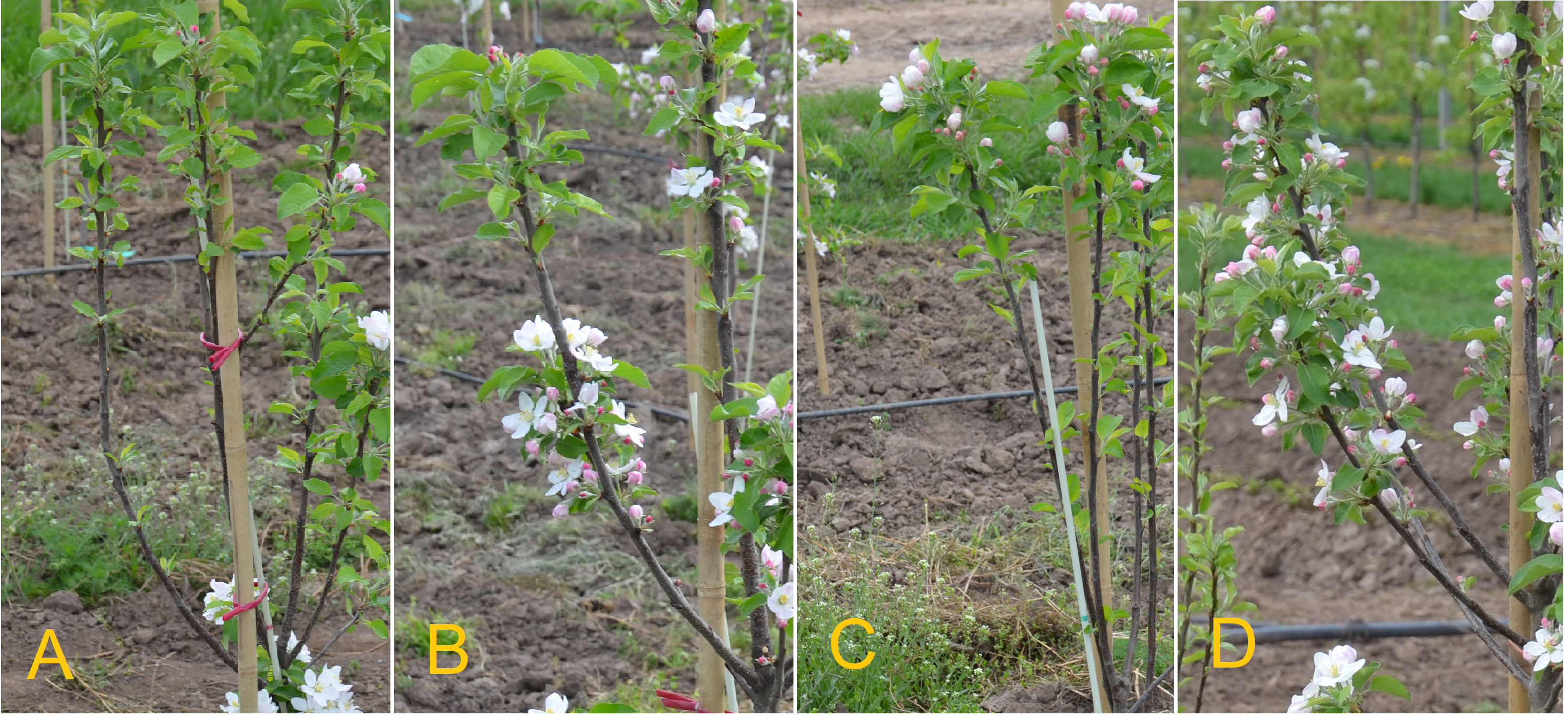

Supplement: S5 Fig — The images show A) no lateral flowers, B) median zone with flower clusters, C) distal zone with flower clusters, and D) median and distal zone with flower clusters as two distinct zones or one continuous zone. (TIFF) [file pone.0285194.s005.tiff]
