# Supplementary material for: Effect of spring nitrogen fertilization on bearing and branching behaviors of young apple trees
Source: PLoS One. 2023 May 4;18(5):e0285194. doi: 10.1371/journal.pone.0285194 (PMC10159155; doi:10.1371/journal.pone.0285194)
Supplement: S1 Table — (DOCX) [file pone.0285194.s006.docx]

| Year | Cultivar | Treatment | Shoot number per tree | | | Lateral shoots length (mm) | | |
| --- | --- | --- | --- | --- | --- | --- | --- | --- |
|  |  |  | SS (min-max) | MS (min-max) | LS (min-max) | SS (min-max) | MS (min-max) | LS (min-max) |
| 2016 | Rubinola | Control | 0.0 | 0.0 | 0.0 | 0.0 | 0.0 | 0.0 |
|  |  | 20 g N/tree | 0.0 | 0.0 | 0.0 | 0.0 | 0.0 | 0.0 |
|  |  | 30 g N/tree | 0.0 | 0.0 | 0.0 | 0.0 | 0.0 | 0.0 |
|  | Topaz | Control | 0.3 (0-2) | 0.2 (0-1) | 0.0 | 20.0 (-) | 110.0 (70-150) | 0.0 |
|  |  | 20 g N/tree | 0.0 | 0.1 (0-1) | 0.0 | 0.0 | 270.0 (-) | 0.0 |
|  |  | 30 g N/tree | 0.3 (0-2) | 0.5 (0-4) | 0.1 (0-1) | 30.0 (20-35) | 150.0 (70-290) | 730.0 (-) |
|  | GD | Control | 0.1 (0-1) | 0.9 (0-3) | 0.4 (0-1) | 40.0 (-) | 150.0 (80-280) | 526.0 (380-630) |
|  |  | 20 g N/tree | 0.6 (0-3) | 0.7 (0-4) | 0.7 (0-4) | 30.0 (10-50) | 180.0 (60-290) | 499.0 (310-690) |
|  |  | 30 g N/tree | 0.3 (0-3) | 1.4 (0-4) | 0.8 (0-2) | 20.0 (10-40) | 130.6 (60-270) | 561.0 (300-820) |
| 2017 | Rubinola | Control | 13.9 (5-32) | 3.6 (0-11) | 8.8 (5-11) | 10.0 (-) | 174.2 (60-290) | 533.7 (300-1050) |
|  |  | 20 g N/tree | 19.0 (8-30) | 2.8 (0-9) | 8.9 (5-14) | 10.0 (-) | 168.6 (60-290) | 455.0 (300-940) |
|  |  | 30 g N/tree | 14.3 (8-27) | 2.8 (0-11) | 9.2 (7-12) | 10.2 (10-50) | 182.1 (60-290) | 469.0 (300-960) |
|  | Topaz | Control | 17.5 (10-24) | 5.1 (1-14) | 6.5 (2-10) | 11.8 (-) | 169.2 (60-290) | 523.7 (300-930) |
|  |  | 20 g N/tree | 15.9 (4-22) | 6.2 (3-9) | 6.2 (2-12) | 11.5 (10-50) | 156.3 (60-290) | 478.3 (300-830) |
|  |  | 30 g N/tree | 17.3 (10-24) | 5.1 (0-10) | 5.5 (2-9) | 12.2 (10-50) | 167.9 (60-290) | 436.4 (300-760) |
|  | GD | Control | 18.3 (12-26) | 9.9 (6-12) | 3.0 (0-7) | 10.0 (-) | 158.2 (60-290) | 440.5 (300-700) |
|  |  | 20 g N/tree | 17.3 (5-26) | 10.8 (4-16) | 3.4 (0-8) | 11.1 (10-30) | 174.8 (60-290) | 436.4 (310-670) |
|  |  | 30 g N/tree | 20.9 (15-28) | 10.9 (3-21) | 1.8 (0-4) | 11.2 (10-50) | 167.5 (60-290) | 401.9 (310-540) |
